# Uncertainty Comparison of Visual Sensing in Adverse Weather Conditions**†**

**DOI:** 10.3390/s16071125

**Published:** 2016-07-20

**Authors:** Shi-Wei Lo, Jyh-Horng Wu, Lun-Chi Chen, Chien-Hao Tseng, Fang-Pang Lin, Ching-Han Hsu

**Affiliations:** 1 National Center for High-Performance Computing, No. 7, R&D 6th Rd., Hsinchu Science Park, Hsinchu 30076, Taiwan; jhwu@nchc.narl.org.tw (J.-H.W.); casper@nchc.narl.org.tw (L.-C.C.); 0903049@nchc.narl.org.tw (C.-H.T.); fplin@nchc.narl.org.tw (F.-P.L.); 2 Department of Biomedical Engineering and Environmental Sciences, National Tsing Hua University, No. 101, Section 2, Kuang-Fu Road, Hsinchu 30013, Taiwan

**Keywords:** vision application, outdoor imaging, visual sensing, flood detection

## Abstract

This paper focuses on flood-region detection using monitoring images. However, adverse weather affects the outcome of image segmentation methods. In this paper, we present an experimental comparison of an outdoor visual sensing system using region-growing methods with two different growing rules—namely, GrowCut and RegGro. For each growing rule, several tests on adverse weather and lens-stained scenes were performed, taking into account and analyzing different weather conditions with the outdoor visual sensing system. The influence of several weather conditions was analyzed, highlighting their effect on the outdoor visual sensing system with different growing rules. Furthermore, experimental errors and uncertainties obtained with the growing rules were compared. The segmentation accuracy of flood regions yielded by the GrowCut, RegGro, and hybrid methods was 75%, 85%, and 87.7%, respectively.

## 1. Introduction

In the summer and during typhoon season, the western coast of Taiwan is particularly vulnerable to flooding, especially during the period between May and October. Every year, abundant rainfall causes numerous deaths and serious damage to the economy [1,2,3,4,5,6,7]. One of the most challenging problems with regard to flood response is the precise localization of flood risk. This task is performed by early warning systems (EWSs) for flood prevention and disaster management. EWSs are extensively applied to mitigate flood risk, and they work by detecting abnormalities and predicting the onset of flooding with remote sensors. They can also provide real-time information during floods [8,9,10,11]. Traditionally, EWSs monitor flooding with remote sensing technology such as satellite imaging and electronic sensors installed nearby rivers and seaports. Satellite images cover hundreds of kilometers, generally providing only the broadest outlines of potential risk. On the other hand, using electronic sensors to measure water levels remains unfeasible, owing to the sheer number of sensors needed. These devices have a limited geographic range and extensive power requirements. Moreover, they incur massive costs in installation and maintenance. Therefore, the development of a long-term sustainable EWS with the ability to precisely localize areas of risk is crucial to the field of flood monitoring and early warning.

Visual sensing techniques are widely employed in various fields for vision applications such as inspection, surveillance, and monitoring. Unlike active sensors, vision sensing techniques indirectly measure physical information from captured images and video. Such systems record particular behavior, activity, and other changes in the field scene. The use of a visual sensing system to perform an indirect estimate of the region, position, velocity, and attitude of a monitored object is well known. However, the influence of weather phenomena on visual sensing systems remains an open research issue with many unanswered questions [12,13]. Conventional imaging systems are designed to capture scenes in ideal atmospheric conditions, such as indoors. However, outdoor vision applications must be capable of capturing images even in adverse weather conditions [14,15]. Such conditions limit the accuracy of the estimated attitude of a monitored object [16,17,18,19].

Fog and stained lenses are the most pernicious phenomena for outdoor vision systems. The image intensity, color, and shape are altered by interactions between light and the atmosphere. First, fog results from suspended particles, mist, raindrops, rain streaks, and heavy spray rain. Another major source with fog are raindrop streaks. Consider a camera system capturing the volume of raindrops; this volume comprises randomly-distributed and high-velocity raindrops. Raindrop streaks are projected in non-uniform stripes onto the scene. They produce sharp changes to the intensity during image acquisition. Subsequent imaging processes are also affected by concentrated rain streaks. Relevant research regarding raindrop detection and removal can be found in [20,21,22,23,24].

**Figure 1:**
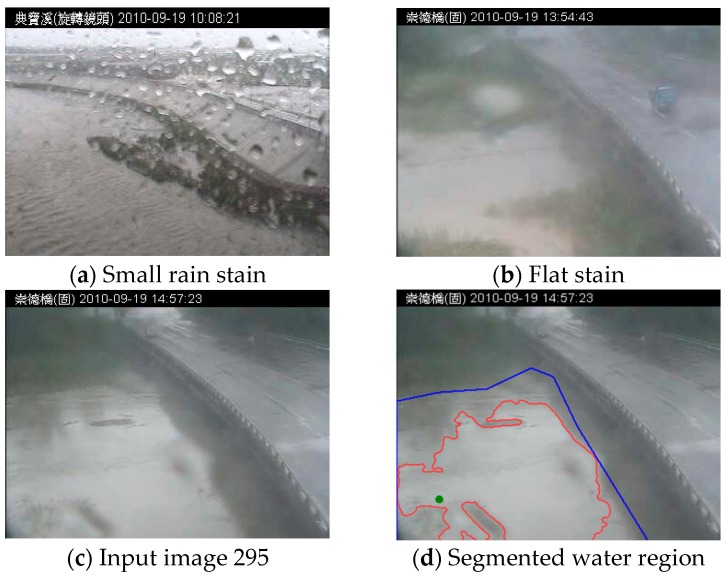
Two stain types: (**a**) a small stain and (**b**) a flat stain overlapping the camera lens. The effect of stains when applied to flood detection for (**c**) a stained outdoor image and (**d**) the concave region affected by stains on an image. Image 295’s flood region segmented with RegGro is represented by a red contour. The blue contour represents the ground truth, and the green dot is the location of the seed used with RegGro. (Note: The Traditional Chinese in header of (**a**) and (**b**–**d**) are represented the location in the Dianbao River and the Changed Bridge, respectively).

Second, raindrop stains tend to adhere to the lens of imaging devices. Each stain refracts and reflects light, generating shape and intensity changes in images. Figure 1a,b shows an example of a stain on a camera lens, and Figure 1c,d shows the detection result of a time-varying flood region. In imaging systems, the projections of a raindrop stain on an image are a non-uniform refraction mask on pixels. Due to the composite raindrop stains, the image intensity is randomly nebulized. However, the effects of raindrop stains on camera lenses have not been thoroughly investigated. This study also focused on rain stains, which are a common atmospheric condition in vision systems.

To recover clear images in adverse weather, associative restoration techniques should be introduced. Fog removal techniques can be applied during preprocessing, before visual sensing applications. Fog removal (or image dehazing) techniques restore image clarity by eliminating the medium effects of fog. The basic principle behind recovering fog-free images involves estimating the transmission of light in the medium of fog scenes, and then eliminating the scattered light caused by the medium, in order to provide a clear image. This topic has been discussed in previous literature [25,26,27,28,29]. However, in long-term video sequencing, image dehazing remains challenging, because it is independent from the atmospheric changes in each frame. Variations in luminosity, the fog level, fog distribution, and light scattering randomly affect video sequencing. A single image clarification filter with constant parameters is insufficient for estimating entire video sequences.

To understand the influence of adverse weather conditions, an image segmentation application has been employed to manipulate videos of rainy conditions captured using an outdoor imaging system. The vision application scenario involved flood-tide detection. Flood regions must be segmented into precise shapes in order to determine hazard levels and provide automatic flood warnings to support EWSs [14].

The remainder of this paper is organized as follows: Section 2 reviews the interactive segmentation problem and the advantages of region-based segmentation. Section 3 describes the two region-based rules and the image set in detail. The experimental results are given in Section 4. Finally, a discussion and conclusions are provided respectively in Section 5 and Section 6.

## 2. Image Segmentation in Environmental Application

This paper focuses on flood detection using small-scale monitoring images to identify the part of flow in a water region, surrounding buildings, and geographic background. However, interference introduced from elements—such as variance to the water region, raindrops on the camera screen, blurred scenes from water atomization, and fierce wind—negatively affects traditional image segmentation methods, such as background subtraction, thresholding, and watershed processing.

Image segmentation has been widely applied in industry and medicine. More recently, the process has been used in environmental object analysis [30,31,32,33,34]. For outdoor images, simple segmentation process parameters, such as threshold values, cannot be established for precise flood region segmentation [35,36,37,38,39,40]. This is because region colors, region shapes, scene illumination, fog distribution, rain, and other atmospheric conditions vary over time. Visual information is somewhat independent between frames in video sequences comprised in a single shot.

Interactive image segmentation schemes with a few simple user inputs provide a better solution for natural images than fully-automatic schemes [41]. First, users indicate the location of the object and background using strokes as markers or seeds. Then, images are initially over-segmented into several small contiguous and perceptually similar regions (or superpixels), using mean shift [42], Bayesian flooding [43], graph-based [44,45], or contour-based [46] methods, among others. Finally, the region-merging stage automatically merges the initial regions with constraints to the boundary, shape, region, and topology [43]. The object is obtained from the background following a merging task. However, most interactive schemes require pre-segmentation to divide the image into small regions. Furthermore, most such schemes require the user to draw the specific shape of the initial markers, in order to fit the location, boundary, and features of the object and background. For flood detection, however, the location, boundary, and features of objects are time-varying. By comparison, both region-based segmentation methods analyzed in this paper do not require pre-segmentation; rather, the user roughly places a few seeds on the flow surface without deliberate selection. Region-based segmentation involves selecting seed points in the region of interest and using an algorithm to grow the region from the seed point according to the seed-pixel intensity and previously set criteria. The seed intensity depends on the pixels in each frame, rather than constrained values. This facilitates the successful deployment of the growing process in various frames with differing intensities. Therefore, region-based segmentation is suitable for time-varying intensity and shape conditions.

Based on the above reasons, region-based image segmentation was selected as the most suitable method for identifying flood regions and estimating the degree of hazard. In addition, region-based image segmentation exhibits properties that increase coupling to the seed location, rather than limit the set intensity. Therefore, temporal shape transformations and size variations to the flood regions can be traced.

## 3. Material and Methods

Region-based segmentation involves the assumption that the pixels within a region possess similar properties, such as color, intensity (gray level), and texture. Based on this, criteria for a similarity test were designed to determine whether neighboring pixels in a region are similar. If a similarity criterion is satisfied, neighboring pixels can be inferred to belong to the same growing region as their neighbors. Similarity criteria are crucial factors that shape growing patterns and result in differing final regions. In this study, we used two region-based algorithms to trace flood regions: RegGro (a modified region-growing algorithm), and GrowCut. The criteria used in the growing process differ between these algorithms. Details for these algorithms are provided in the following sections.

### 3.1. RegGro Method

The purpose of RegGro is to group pixels into meaningful regions, starting from a specific seed pixel and spreading to neighbors that satisfy the growing rule [40]. The growing rule is a set of criteria used to determine whether neighboring pixels should be added to the region. The fundamental disadvantage to intensity-based region segmentation is that the intensity provides no spatial information. The established threshold is a single value or a set gray level. Hence, to implement the growing rule, RegGro uses the dynamic mean intensity with a threshold window (where the window size is ± the intensity distance). The dynamic mean intensity is the sum mean intensity of all pixels that belong to a specific region. This mean intensity is updated each time a new pixel is added to the region. Specifically, the mean intensity is a dynamic statistic that depends on the current region, rather than the established intensity of the initial seed pixel. Thus, the mean intensity is more suitable for spreading over the blurred boundary when the region and background have not been determined. The RegGro rule pseudocode can be described as follows (Algorithm 1):
**Algorithm 1: RegGro rule pseudocode.**//For each pixel pfor all p in image A //Copy previous state of p and the mean intensity of all q labels_new=labels; Mean_Intensity(q); //All current q try to spread to neighbors p for all neighbors of p  if(Intensity(p)∈Mean_Intensity(q))     //Successful growth spreads out to the current p.   labels_new(p)=labels(q)  end if end forend for

**Figure 2:**
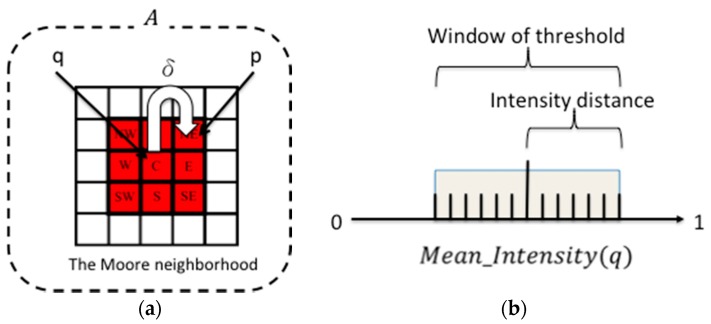
(**a**) Region-growing process from q to 8-connected neighbors p that satisfy the δ function; and (**b**) threshold for the window of intensity. The center of the window is the value of Mean_Intensity, and the window size is ± the intensity distance (±0.065).

Here, *p* represents the set of background pixels. Before segmentation, then, *p* denotes all of the pixels in an image. The image segmentation process can be understood as a process that partitions p into two subregions: the foreground and the background. Initially, all p in the image are labeled as the background region, where q—the pixels belonging to the foreground—are seeded pixels. Initially, the mean intensity of all q, Mean_Intensity(q), is the intensity of only one seeded pixel. This seeded pixel is the first q. Then, *q* attempts to spread to the neighbors (8-connected pixels). The region-growing process involves labeling a neighboring pixel p as the foreground in a larger region when intensity(p) falls within the Mean_Intensity(q), as shown in Figure 2a. The region-growing or spreading rule of the foreground region’s pixel q and neighbors  p, hereafter referred to as the δ function, is defined as follows:

(1) $$p\left(x,y\right)=\{\begin{array}{c} Foreground\;if\;Intensity\left(p\right)\in Window\langle Mean\_Intensity\left(q\right)\rangle \\ Background\;otherwise\; \end{array}$$

where the intensity of pixel p is the V-channel value of an image in the HSV color space. The Mean_Intensity is the pixel mean intensity of q with a window of the intensity distance. This is dynamically updated with each new pixel added to q. The constant intensity distance is a window of the Mean_Intensity set to 0.065, because all images are converted to a floating format ranging between 0 and 1, as shown in Figure 2b.

### 3.2. GrowCut Method

**Figure 3:**
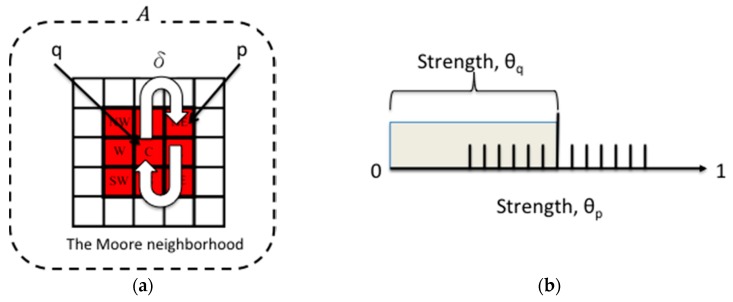
(**a**) Region-growing process from cell q to its neighbors or reverse-growing from its neighbors, with the δ function; and (**b**) the strength threshold for the growing rule. The region grows when $\theta_{q}>\theta_{p}$; otherwise the region reverse-grows.

GrowCut provides an alternative to region-based methods. GrowCut applies cellular automation as the region-growing rule [47]. In automata evolution models, each pixel is treated as a cell that grows and struggles with other cells. Region growing begins from the seed pixels, spreads outward, and attempts to occupy the entire image. Here, the region growth criteria are called the local transition function, known as the δ function. This function defines the rule for calculating the state of a current cell coupled with the state of neighboring cells. Moreover, unlike traditional region growing in only one direction, the state of the region pixels can reverse-grow with neighboring pixels. Thus, the automation evolution can grow the region bi-directionally until all criteria have been satisfied (Figure 3). The pseudocode of the automata evolution rule is described as follows (Algorithm 2):
**Algorithm 2: GrowCut rule pseudocode.**//For each cell pfor all p in image A //Copy the previous state of p $l_{p}^{t+1}=l_{p}^{t};\theta_{p}^{t+1}=\theta_{p}^{t};$ //All current cells q try to attack p  for all neighbors p  if $g\left(\|\vec{C_{p}}-\vec{C_{q}}\|\right)\cdot \theta_{q}^{t}>\theta_{p}^{t}$
   //Successful attacks spread to neighbors p   $l_{p}^{t+1}=l_{q}^{t};\theta_{p}^{t+1}=g\left(\|\vec{C_{p}}-\vec{C_{q}}\|\right)\cdot \theta_{q}^{t};$  end if end forend for
where the label $l_{q}$ denotes a foreground pixel, label $l_{p}$ denotes a background pixel, θ is the strength of the pixels, and θ∈[0,1]. Here, $\vec{C}$ is the intensity of the pixel, and $g\left(\|\vec{C_{p}}-\vec{C_{q}}\|\right)$ is the absolute difference between p and q. In the initial states, all q are set to $l_{q}=0\;\left(0=background,\;1=foreground\right),\;\theta_{q}=0,\;\vec{C_{q}}=Seed\left(x,y\right)$. The growing rule for GrowCut—i.e., the δ function—is defined as follows:

(2) $$p\left(x,y\right)=\{\begin{array}{c} Foreground\;if\;g\left(\|\vec{C_{p}}-\vec{C_{q}}\|\right)\cdot \theta_{q}^{t}>\theta_{p}^{t}\\ Background\;Otherwise\; \end{array}$$

### 3.3. Hybrid RegGro and GrowCut

We proposed a hybrid RgGc that employs a neural network model in order to combine these two growing methods. The hybrid RgGc applies a neural network to classify the input image as fog, stained, or normal scenes. Then, RegGro and GrowCut were applied to process fog and stain images, respectively. The GrowCut method has also been used to segment images of normal scenes.

Detecting fog and stain scenes is a difficult task for image recognition. It is also unclear how the properties of fog and stain should be described. Currently, neural networks have been central to the largest advances in image recognition performance in recent years. The network model learns what distinguishes images, rather than relying on manually-specified differences. To automatically recognize the fog and stain images, a neural network model is presented as a classifier. This model is trained using TensorFlow [48]. Following the training instruction [49], the model is trained with the Typhoon Image Set, as described in Section 3.4, to distinguish between three labels (viz., fog, stained, and normal). This model uses 4000 training steps. Each step chooses ten random images from the Typhoon Image Set, and feeds them into the final layer in order to derive predictions. Those predictions were then compared to the actual labels in order to update the final layer's weights through the back-propagation process. This test evaluation is the best estimate of how the trained model will perform with regard to the classification task. Model evaluations were performed using a running average of the parameters computed over time. After the model was fully trained, its accuracy was approximately 99%.

**Figure 4:**
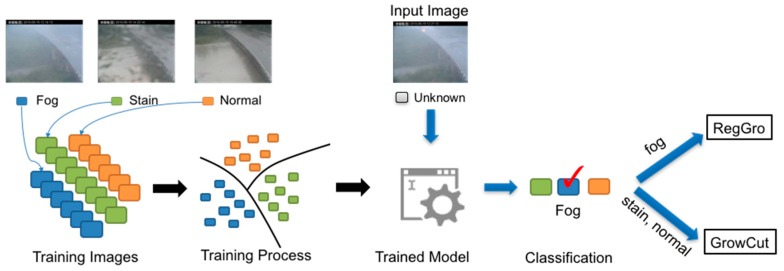
Flowchart of the training model and classification input images. During the training process, the training images are labeled as fog, stained, or normal. The hybrid RgGc method classifies input images and then pipes to different growing methods. (Note: The Traditional Chinese in header of all images is represented the location in the Changed Bridge).

Figure 4 shows the workflow of the training model. The trained model classifies an input image. A decision is made regarding whether an input image is foggy, stained, or normal. The hybrid RgGc then automatically switches to the RegGro and GrowCut methods to process fog and stained images separately.

### 3.4. Image Set and Ground Truth

#### 3.4.1. Typhoon Image Set

**Figure 5:**
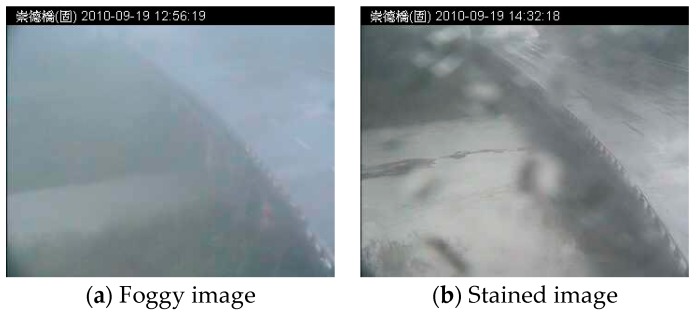
Image set and weather conditions. The image set was captured in adverse weather conditions. The selected sample images show fog (**a**) and stained (**b**) patterns. (Note: The Traditional Chinese in header of all images is represented the location in the Changed Bridge).

In this case study, two region-based segmentation algorithms were employed to identify flood regions. Historical outdoor images were recorded during a typhoon-induced rainstorm that occurred in September 2010 in Taiwan. The capture period was between 12:00 p.m. and 5:50 p.m., 19 September 2010. The outdoor imaging system replayed real-time videos streamed to Internet applications. For our evaluation, we extracted one image each minute, for a total of 350 images in the test image set. The video stream was decomposed to a spatial resolution of 352 × 288 in JPEG format. Part of the test image set is shown as thumbnails in Figure 5. The images were captured between noon (when the raining began) and nightfall (at the flood tide).

#### 3.4.2. Ground Truth of Flood Segments

**Figure 6:**
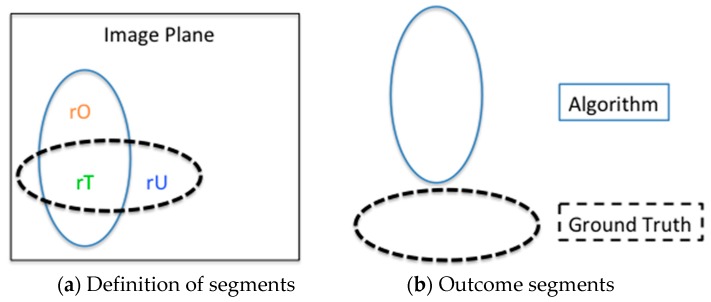
Accuracy determined according to the ground truth. (**a**) the resulting region pixels of the algorithm in the image plane are classified as: rT, matching the ground truth; rO, over-segmented; and rU, under-segmented; (**b**) the outcome segments produced with algorithm and ground truth.

**Figure 7:**
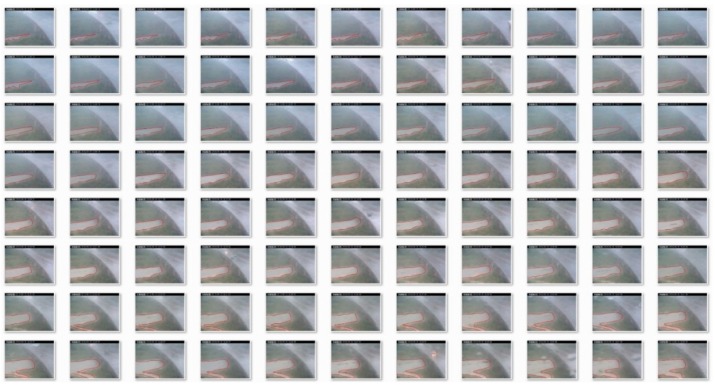
Part of the ground truth of the image set. The red boundaries are manually-labeled segments of flood regions. (Note: The Traditional Chinese in header of all images is represented the location in the Changed Bridge).

To evaluate the segmentation results of previous algorithms, a ground truth that yields accurate segmentation results is needed. The ground truth also provides a statistical basis for evaluating region segmentation and boundary detection, as shown in Figure 6. Therefore, flood regions in 450 outdoor images were labeled manually. Examples of these manually-labeled flood regions are shown in Figure 7, where the red boundaries represent the flood region coverage in the original images. The “true detection” and “false detection” of detected flood segments in each image are described as follows:

(3) if {(rT>70% of g.t.)&&(rO<30% of g.t.)&&(rU<30% of g.t.)}; True Detection else Fault Detection

where, *g.t.* denotes the pixels of the ground truth, *rT* is the resulting region pixel of the algorithm that matches the *g.t.*, *rO* denotes over-segmenting that grows to non-*g.t.*, and *rU* denotes under-segmenting that misses the *g.t.*

The algorithms’ respective accuracy for the whole image set is derived as follows:

(4) Algorithms’ Accuracy= (∑​TrueOfDetection∑​ImageSet)× 100%

## 4. Results

RegGro and GrowCut were employed to determine flood regions in outdoor images. Various seed-location and image-filtering settings were tested to determine the optimal set that resulted in superior flood regions. The results of flood segments were evaluated according to the ground truth.

### 4.1. Performance of RegGro

**Figure 8:**
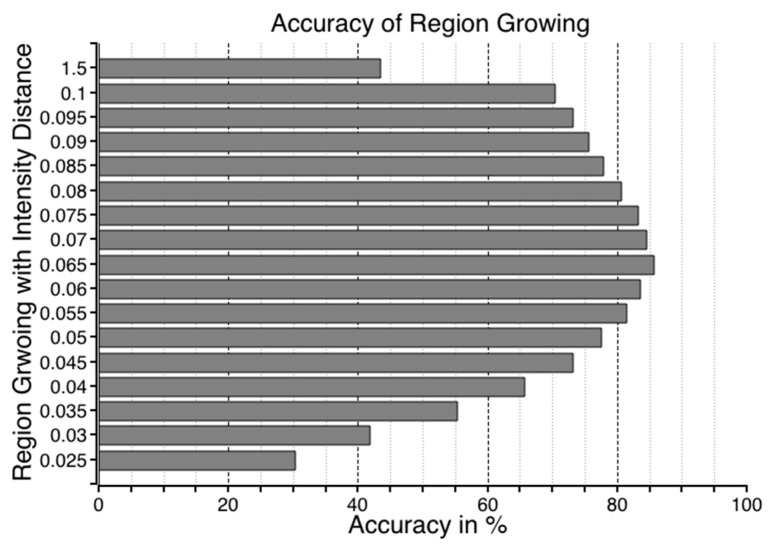
RegGro accuracy. The accuracy was determined according to the ground truth. Each horizontal bar shows the accuracy with a different intensity distance, ranging from 0.025 (RegGro_025) to 0.15 (RegGto_150). The highest accuracy was 85.7% with an intensity distance of 0.065.

**Figure 9:**
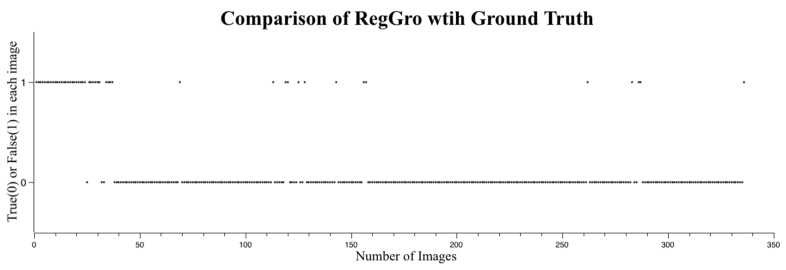
Segmentation success or failure with RegGro. True (0) indicates success, and False (1) indicates failure. Most false detections occurred in the first 40 images with heavy rain and fog.

**Figure 10:**
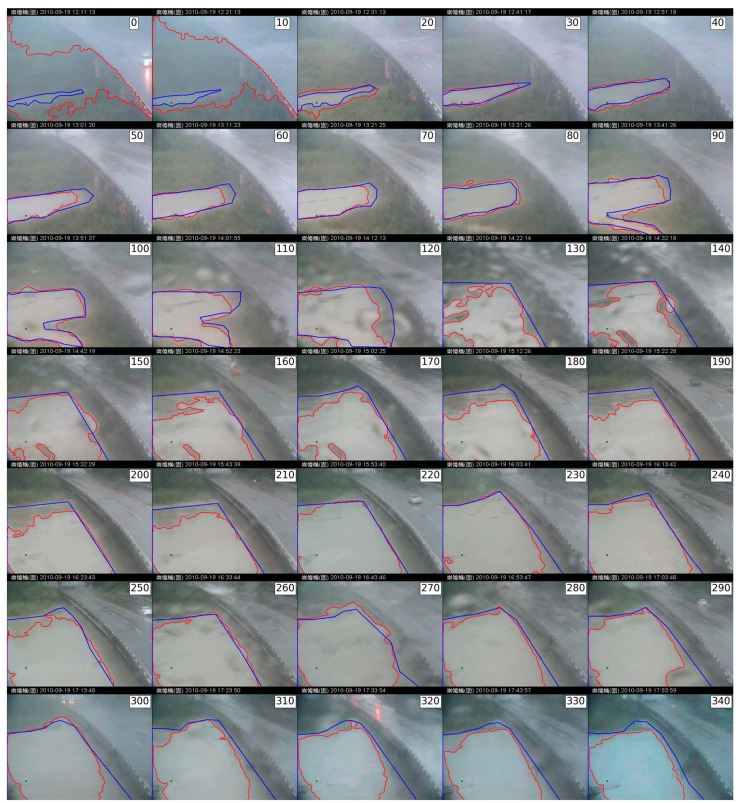
Part of RegGro’s results with red segments from growing methods. The blue line is the ground truth, and the green marker is the initial seed for the growing methods. There were few flood segmentation failures in heavy rain and fog. Some failures occurred with raindrop stains on the CCTV screen. (Note: The Traditional Chinese in header of all images is represented the location in the Changed Bridge).

The accuracy of the flood regions identified with RegGro is shown in Figure 8. The intensity distance ranged from 0.025 to 0.15. The highest accuracy achieved using RegGro was 85.7%, with an intensity distance of 0.065. Images without flooding were excluded to avoid the problem of selecting seed points in non-object regions. The remaining 335 valid images were used to evaluate the segmentation algorithms. In a prior experiment, we found that image filtering cannot substantially improve the segmentation accuracy of the RegGro algorithm. We examined several image filters, including the mean, median, bottom-hat, and histogram equalization. However, the maximum accuracy of RegGro was achieved using non-filtered images. To thoroughly understand the segmentation performance, the comparison results of image sequences are presented in Figure 9. The data in Figure 9 show the flood region accuracy evaluated within a time series. This process is crucial for an EWS in order to trace flood variations precisely during the tide process. Inconsistent segments were set as False (1), and consistent segments were set as True (0). This clearly indicates that the segmentation accuracy for the initial period of rain was insufficient. Specifically, before Image 40, the majority of flood segments were not consistent with the ground truth. The remaining flood segments exhibited accurate regions, excluding a few failures in subsequent images. The results of the flood-region segmentation are partially shown in Figure 10 with a step of 10 frames.

### 4.2. Performance of GrowCut

**Figure 11:**
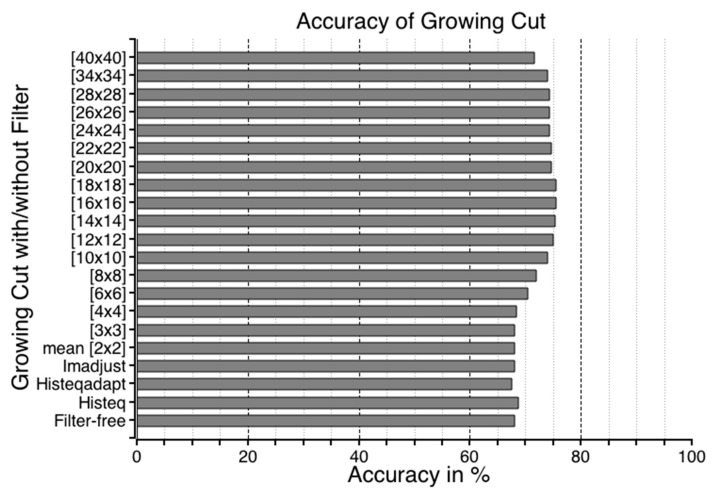
GrowCut accuracy. The accuracy was determined according to the ground truth. Each horizontal bar shows the accuracy with a different filter: viz., the mean, imadjust, histeqadapt, histeq, and filter-free GrowCut. The highest accuracy is 75.2% with the mean filter and both 18 × 18 and 16 × 16 masks.

**Figure 12:**
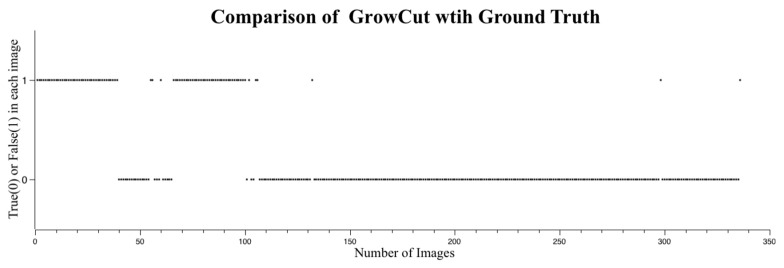
Segmentation success or failure with GrowCut. True (0) indicates success, and False (1) indicates failure. Most false detections occurred between Images 0–40 and Images 70–100 with heavy rain and fog. However, GrowCut was more robust to raindrop stains on the CCTV screen (after Image 100).

**Figure 13:**
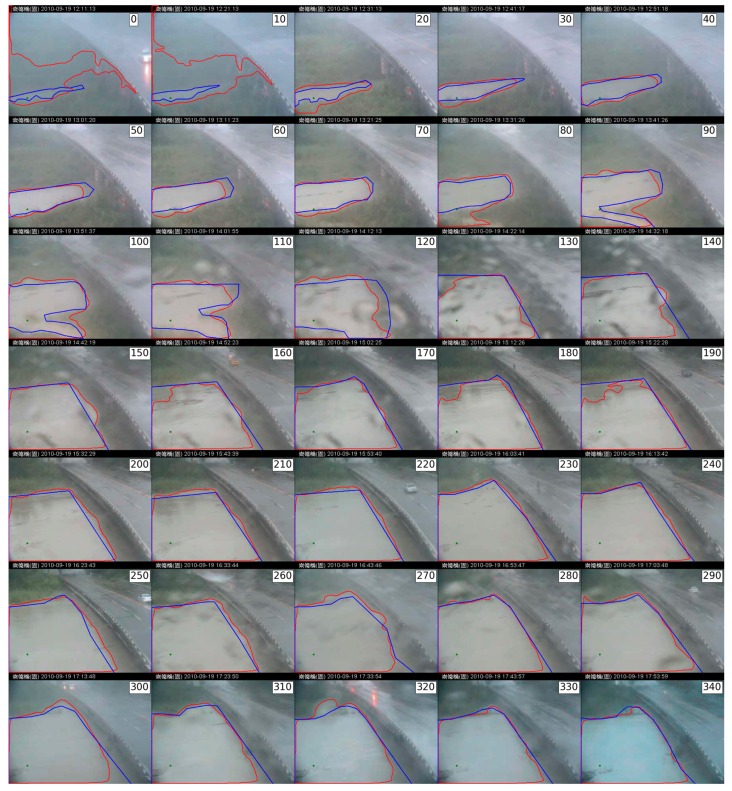
Part GrowCut’s results with red segments from growing methods. The blue line is the ground truth, and the green marker is the initial seed for the growing methods. Most flood segmentation failures occurred during heavy rain and fog. GrowCut is robust to raindrop stains on the CCTV screen. (Note: The Traditional Chinese in header of all images is represented the location in the Changed Bridge).

The accuracy of flood regions identified with GrowCut is shown in Figure 11. Unlike RegGro, some image filters in GrowCut can improve flood detection in various segments. The maximum accuracy was 75.2% when using the mean filter with 16 × 16 or 18 × 18 masks. After testing several image filters, the experimental results showed that the mean filter is superior for enhancing the outcome provided by GrowCut. Specifically, the mean filter increased the accuracy of the GrowCut algorithm from 68.1% to 75.2%. To thoroughly understand the segmenting performance, a comparison of image sequences is shown in Figure 12. The data in Figure 12 indicate the flood region accuracy evaluated within a time series. Inconsistent segments were set to False (1), and consistent segments were set to True (0). This clearly indicates that the segmenting accuracy for the initial rain period failed during two periods of rain. The first period was the same as that using RegGro. That is, before Image 40, most flood segments were not consistent with the ground truth. The second period was between Images 70 and 100, and exhibited more failed segments than RegGro. In the remaining flood segments, however, GrowCut yielded only a few failures in subsequent images. In other words, GrowCut provided nearly perfect segmentation from Image 100 onward. The results of flood region segmentation are partially shown in Figure 13 with a step of 10 frames.

### 4.3. Performance of Hybrid RegGro and GrowCut

When combining RegGro and GrowCut, the hybrid RgGc was 87.7% accurate. The accuracy of the flood regions identified with the hybrid RgGc is shown in Figure 14. To thoroughly understand the segmentation performance, the comparison results of image sequences are presented in Figure 14. The data in Figure 15 show the flood region accuracy evaluated within a time series. Inconsistent segments were set as False (1), and consistent segments were set as True (0). This result also clearly indicates that both RegGro and GrowCut failed to segment the flood regions as well as the hybrid RgGc during the initial period of heavy rain and fog. The results are consistent with the previous observations in Section 4.2 and Section 4.3. The hybrid RgGc exploited the strength of both growing methods, with more accurate detections than GrowCut for Images 65~100, and RegGro for Images 110–150. The results of the flood-region segmentation, ground truth, and seed marker are partially shown in Figure 16.

## 5. Discussion

In this section, we discuss how poor atmospheric conditions affect segmentation outcomes and ground-truth proceedings. As stated in the introduction, fog and stains are the primary factors that affect the outcome. Moreover, the ground truth also serves as a crucial evaluation factor.

### 5.1. Influence of Fog and Heavy Rainfall

Fog and haze are the main factors that disturb light reflection in scenes, causing unexpected variations in image intensity. In our image set, fog and haze occurred for only a short period when the rainstorms began—specifically, the period between Images 0 and 40. Since rainstorms involve suspended particles, mist, raindrops, raindrop streaks, and heavy rain spray, they render scenes extremely unclear. An image of the fog that formed before the initial rainstorm at noon is shown in Figure 17. The resulting blurry image was difficult to segment using the proposed region-based method. We enhanced the image using filters and equalization to improve the histogram distribution. However, this enhancement procedure also affected the remaining images, causing the region-based segmentation method to tend toward overestimations and underestimations. Both algorithms failed to segment the initial period of torrential rainfall. Therefore, we infer that the presence of fog and haze influence the final outcome of flood segmentation. Furthermore, during heavy rainfall, CCTV cameras that rely exclusively on visible light are more easily blocked by raindrops and fog. Multispectral image sensors should be used to address this issue [50]. For example, infrared cameras use infrared light to capture scenes. Infrared light has a longer wavelength than visible light, and it can penetrate heavy fog and rainfall to form clearer images.

### 5.2. Influence of Stains on Lenses

Various stains on the camera lens also exert critical effects on image processing. Figure 18 shows an example of the differing outcomes of these algorithms with these effects. Briefly, image segmentation is a process that involves segmenting objects of interest from the background. Although extracted segments have discriminatory boundaries, they are sensitive to minor foreground and background boundaries. However, the presence of stains on the camera lens directly disturbs the overall image intensity. Occasionally, the segmentation process stalls on stained areas (see Image 270 in Figure 18b). This is the reason why RegGro yielded numerous segmentation failures from Images 100–350 (see Figure 9). GrowCut exhibited a superior ability to resist rain stains on the camera lens during the specific period between Images 100 and 350 (Figure 12). We compared crucial periods, at the start (Image 152) and middle (Image 270). Although RegGro provided superior segmentation accuracy during the initial period, the effects of stained areas stalled region growth. By contrast, GrowCut exhibited superior resistance to stains on the camera lens (see Image 270 in Figure 18a), yet it tended to overestimate flood regions at the start (see Image 152 in Figure 18a). Thus, GrowCut primarily yielded segmentation failures before Image 100 (see Figure 12).

### 5.3. Ground Truth

We also inquired as to whether a ground truth is the only method for estimating the performance of an algorithm. However, it must be asked whether the ground truth exactly represents the flood region. In Figure 19, compared with the ground truth segment, the RegGro segment determines segmentation failure. In this case, the RegGro segment is not considered a flood region, ostensibly leading to a false detection. In fact, this RegGro segment can be treated as an assembly of various flood regions. Moreover, manually-labeled flood regions are sometimes underestimated, because the subjects sedulously avoid solid boundaries in order to ensure that segments remain isolated from the background. Thus, the manually-labeled region might be smaller than the actual region. To evaluate the performance success or failure, an intelligent and flexible evaluation of the ground truth should be conducted in future studies.

## 6. Conclusions and Future Work

Vision systems are used for various image applications, such as feature detection, stereo vision, segmentation, recognition, and tracking. These image processes are affected by weather conditions, particularly fog and stains that occur on lenses. However, such visual effects barely affect human vision, because the original scene behind the stain can, nevertheless, be approximated. In most image processing applications—particularly processes that use the pixel intensity and graph information—stained regions belong to neither the foreground nor the background. Therefore, outdoor imaging and monitoring applications are limited by the visual effects generated by adverse weather conditions. This was the primary motivation for this study. We aimed to understand not only the influence of adverse weather on outdoor imaging, but also how the performance of image applications can be improved.

**Figure 15:**
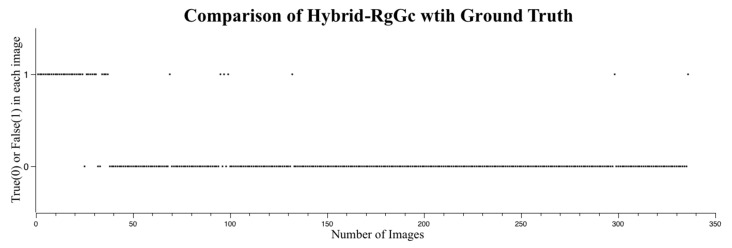
Segmentation success or failure with the hybrid RgGc. True (0) indicates success and False (1) indicates failure. Most false detections occurred in the first 40 images with heavy rain and fog. Both methods failed to segment the flood regions as well as the hybrid RgGc.

**Figure 17:**
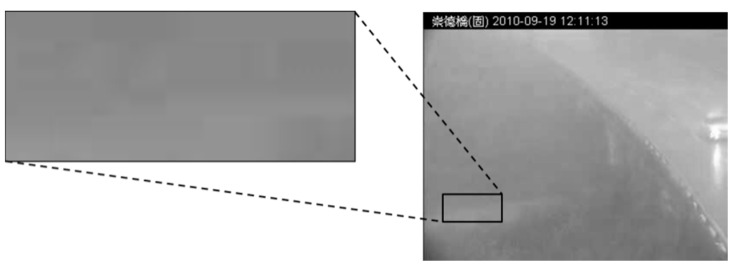
Fog example. Fog and haze regress pixel intensity. (Note: The Traditional Chinese in header of all images is represented the location in the Changed Bridge).

**Figure 18:**
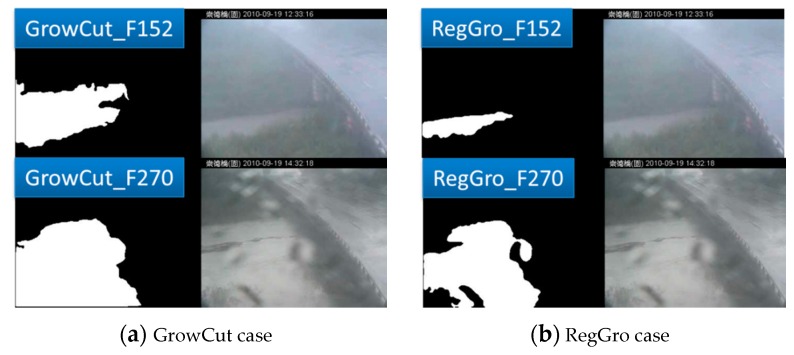
Comparison of the two algorithms with example images from the start (Image 152) and middle (Image 270). (**a**) GrowCut covering the stains (Image 152) and overestimating them (Image 270); (**b**) RegGro identifying the flood region (Image 152) and affected by stains (Image 270). (Note: The Traditional Chinese in header of all images is represented the location in the Changed Bridge).

**Figure 19:**
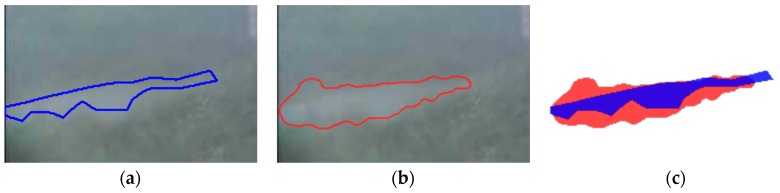
Example of a failed identification. The RegGro region (in red) overlaps with the ground truth (in blue). (**a**) ground truth; (**b**) RegGro region; and (**c**) comparison of regions.

In this study, two region-based segmentation algorithms and a hybrid method that combines both algorithms were applied to flood detection in adverse weather. A case study was presented to demonstrate the performance of these algorithms and their bottleneck for low-cost vision-based flood monitoring. The experimental results indicate the advantages and disadvantages of both algorithms, and the effects that poor atmospheric conditions have on segmentation outcomes. Both methods have unique advantages and disadvantages for fog and stained conditions, respectively. The segmentation accuracy of flood regions yielded by GrowCut and RegGro was 75% and 85%, respectively. Although RegGro was more accurate, it was inadequate for stained images. If the ability to resist stains were incorporated into RegGro, it could achieve more accurate results (see Figure 15 for the hybrid’s results). Thus, we have combined the advantages of both RegGro and GrowCut into a hybrid RgGc with a network classifier. In doing so, we improved the results by approximately 2.7%. Moreover, we shall investigate the feasibility of multispectral cameras in terms of improving the accuracy of image segmentation and preserving visual information in outdoor images with scenes of fog.

## Figures and Tables

**Figure 14:**
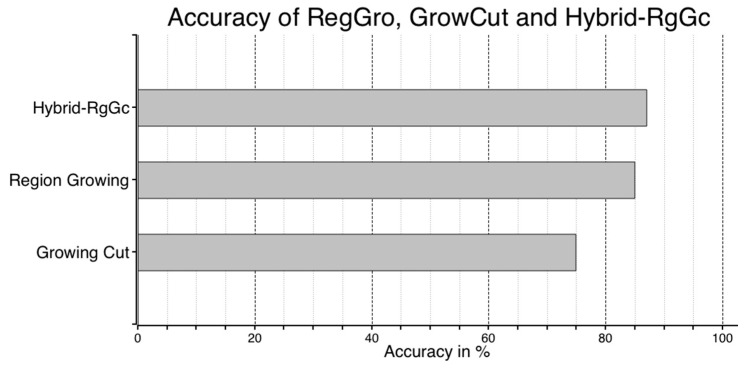
Hybrid RgGc accuracy. The accuracy was determined according to the ground truth. Each horizontal bar shows the accuracy with a different growing method. Outperforming both RegGro and GrowCut, the hybrid RgGc was 87.7% accurate.

**Figure 16:**
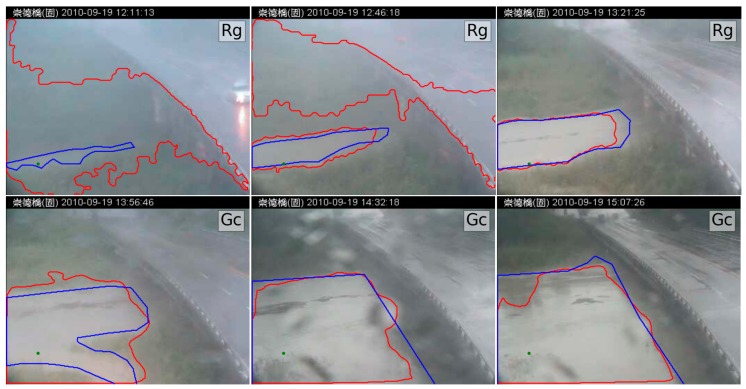

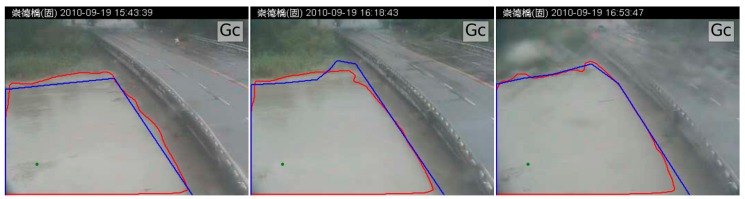
Hybrid RgGc results with red segments from growing methods. The blue line is the ground truth, and the green marker is the initial seed for the growing methods. This figure only shows Images 0, 35, 70, 105, 140, 175, 210, 245, and 280 from the image set, in order to clearly show the contours and seeded marker. The text in the upper-right corner distinguishes between images processed with RegGro (Rg) and GrowCut (Gc). (Note: The Traditional Chinese in header of all images is represented the location in the Changed Bridge).
